# The reliability and quality of short videos as a source of dietary guidance for cardiovascular disease: a cross-sectional study

**DOI:** 10.3389/fcvm.2026.1878079

**Published:** 2026-07-29

**Authors:** Ruixue Song, Mingjie Wang, Shuzheng Yu, Ting Liu, Xisha Liao, Hongmei Zhang, Liyun Zhang, Ziwei Liao

**Affiliations:** 1 Department of Emergency Medicine, Qilu Hospital of Shandong University, Jinan, China; 2 Shandong Provincial Clinical Research Center for Emergency and Critical Care Medicine, NMPA Key Laboratory for Clinical Research and Evaluation of Innovative Drug, Medical and Pharmaceutical Basic Research Innovation Center of Emergency and Critical Care Medicine, China’s Ministry of Education, Shandong Provincial Engineering Laboratory for Emergency and Critical Care Medicine, Key Laboratory of Cardiopulmonary-Cerebral Resuscitation Research of Shandong Province, Qilu Hospital of Shandong University, Jinan, China; 3 Department of Neurosurgery, Ganzhou Hospital-Nanfang Hospital, Southern Medical University, Ganzhou, Jiangxi, China; 4 Department of Neonatology, Ganzhou Hospital-Nanfang Hospital, Southern Medical University, Ganzhou, Jiangxi, China; 5 Department of Emergency, Ganzhou Hospital-Nanfang Hospital, Southern Medical University, Ganzhou, Jiangxi, China; 6 Department of Emergency, The Fifth People’s Hospital of Ganzhou, Ganzhou, Jiangxi, China

**Keywords:** accuracy, cardiovascular disease, diet, Douyin, nutrition, quality, short videos

## Abstract

**Background:**

Short-form video platforms are increasingly used as sources of health information. However, the accuracy and trustworthiness of cardiovascular disease (CVD)-related dietary guidance remains unclear. This study aimed to evaluate the quality, reliability, and content characteristics of CVD-related dietary guidance videos on Douyin, the Chinese version of TikTok.

**Methods:**

A cross-sectional study was conducted on April 7, 2026, using Douyin as the data source. A total of 98 videos directly related to CVD dietary guidance were included after screening. Baseline characteristics, uploader types, and user engagement metrics were extracted. Video quality and reliability were independently assessed using the Global Quality Score (GQS), the Journal of the American Medical Association (JAMA) benchmark criteria, and the modified DISCERN (mDISCERN) instrument.

**Results:**

Among the 98 included videos, 59.0% were uploaded by professional individuals and 11.2% by professional institutions. Fruits and vegetables (98.0%) and grains and tubers (87.0%) were the most frequently covered dietary categories, whereas oil and salt restriction was less frequently addressed (47.0%). Videos uploaded by professional institutions and medical professionals achieved higher GQS, mDISCERN, and JAMA scores than those from nonprofessional creators (all *P* < 0.001). Video quality showed weak positive correlations with engagement metrics, including followers and likes (Spearman r = 0.24–0.31, all *P* < 0.05). GQS, mDISCERN, and JAMA scores were positively correlated (Spearman r = 0.35–0.40, all *P* < 0.001).

**Conclusion:**

Douyin videos provide accessible dietary guidance for CVD, but overall quality and comprehensiveness remain suboptimal. Professional involvement improves reliability and evidence-based content. Regulatory oversight, standardized quality criteria, and targeted education for content creators are needed to ensure accurate dietary information dissemination.

## 1 Introduction

Cardiovascular disease (CVD) remains one of the leading causes of mortality and disability worldwide, posing a substantial and persistent burden on global public health systems (1). Recent projection studies indicate that the global prevalence of CVD is expected to nearly double, increasing from 598 million cases in 2025 to approximately 1.14 billion by 2050, with an annual growth rate of 3.6% (2). Correspondingly, the number of deaths attributable to CVD is projected to rise markedly, from 20.5 million in 2025 to 35.6 million by 2050, representing a 73.4% increase (2). In China, the situation is particularly concerning; with accelerated population aging and rapid lifestyle transitions, CVD has become the leading cause of death among both urban and rural residents, accounting for over 40% of all-cause mortality (3). A substantial body of high-quality evidence has consistently demonstrated that dietary management is a cornerstone of CVD prevention and an essential adjunct to clinical treatment (4–6). Current national and international guidelines widely recommend dietary patterns rich in whole grains, fruits, vegetables, nuts, and high-quality protein sources, while emphasizing the restriction of sodium, sugar-sweetened beverages, and saturated fats to effectively reduce cardiovascular risk (7–9). Despite these well-established recommendations, significant gaps persist between patients' knowledge of dietary strategies and their actual implementation in daily life (10–13). Traditional health education approaches—such as outpatient counseling, printed educational materials, and professional medical literature—are often limited in accessibility, scalability, and long-term engagement (10, 14). In recent years, the rapid advancement of internet and mobile technologies has fundamentally transformed the ways in which individuals access health information. This trend has been further accelerated by the COVID-19 pandemic, during which reliance on online health resources has increased substantially (15). It has been reported that more than 80% of patients have sought health-related information via the internet (16).

Users' engagement with health-related short videos is influenced by multiple psychological and platform-related factors. Perceived source credibility, professional identity cues, emotional framing, and social endorsement (e.g., likes and comments) may enhance trust and engagement, while algorithmic recommendation systems reinforce repeated exposure and perceived familiarity (17, 18). Conversely, lack of professional attribution or inconsistency with established medical guidelines may reduce credibility (17, 19). Evidence suggests that exposure to online health information can influence health-related behaviors, including dietary choices, medication adherence, and healthcare-seeking decisions (20, 21). Therefore, assessing the quality of short-video health content is essential given its potential impact on real-world behaviors.

With continuous innovations in information dissemination and storage technologies, media formats have undergone significant evolution (22). Short-video platforms, particularly Douyin (the Chinese version of TikTok), have emerged as dominant channels for health communication. Compared with traditional text-based information, audiovisual content is more engaging, easier to comprehend, and more likely to be retained, thereby facilitating patients' ability to evaluate their health status and adopt appropriate dietary behaviors (23, 24). These platforms allow users to access a vast amount of health-related content simply by entering keywords, without requiring registration or payment. Consequently, an increasing number of healthcare professionals, institutions, and lay content creators are disseminating disease-related knowledge through short videos, including dietary guidance for CVD. However, for most patients, assessing the quality and reliability of online dietary information remains challenging (25, 26). Importantly, a considerable proportion of health-related videos are produced by non-professional individuals lacking formal medical training, which raises concerns regarding misinformation, bias, and potential harm to public health (27–29). To address these issues, several standardized tools have been developed to evaluate the quality and reliability of online medical content, including the Journal of the American Medical Association (JAMA) benchmark criteria, the modified DISCERN instrument, and the Global Quality Score (GQS). These tools have been widely applied in assessing short-video content related to specific cardiovascular conditions, such as myocardial infarction, coronary artery disease, and pulmonary arterial hypertension (30–32).

To date, however, there has been a lack of systematic evaluation of the quality and reliability of short videos focusing on dietary guidance for patients with CVD on Douyin. Therefore, this study aims to comprehensively assess the baseline characteristics, uploader profiles, content types, user engagement metrics, and quality scores of such videos. By doing so, we seek to provide an objective evaluation of the credibility of diet-related CVD information on short-video platforms, thereby informing clinicians, health educators, and policymakers about the role and implications of social media in contemporary health communication.

## 2 Methods

### 2.1 Study design and data source

A cross-sectional content analysis was conducted to evaluate the informational quality and content characteristics of short videos related to dietary guidance for cardiovascular disease on Douyin (the Chinese version of TikTok). Video retrieval was performed on April 7, 2026. To minimize algorithm-driven personalization, a newly registered account was used, and all browsing history was cleared prior to data collection. The predefined combined Chinese search query was “心血管疾病 膳食营养”, which translates as “cardiovascular disease, dietary nutrition”. The term predefined indicates that the search terms were determined before data collection based on the study objective and were not modified after reviewing search results. The sample size was predetermined, and the first 100 videos under the platform's default ranking were screened sequentially. This approach aligns with prior short-video content analyses that use top-ranked results to reflect user exposure. The choice of 100 videos also balanced feasibility and detailed manual quality assessment using multiple validated instruments, and is consistent with previous studies (33, 34). The exact platform's default ranking algorithm is proprietary and not publicly disclosed; however, it likely incorporates relevance to the search query, user engagement, popularity, recency, and platform recommendation mechanisms.

### 2.2 Eligibility criteria

Videos were included if they: (1) primarily addressed dietary guidance for cardiovascular disease; (2) were publicly accessible; (3) were presented in Chinese; and (4) contained sufficient audiovisual information for evaluation.

Videos were excluded if they: (1) consisted mainly of advertisements or promotional content; (2) were duplicated videos with identical or highly similar content; or (3) had durations shorter than 15 s, as such videos usually contained insufficient substantive information for quality assessment; (4) had durations longer than 10 min, as these more closely resembled long-form lectures or recordings rather than typical short-video content.

### 2.3 Data extraction and variable definition

Two reviewers independently extracted video-level data using a standardized form. Extracted variables included uploader characteristics (uploader type and professional background), engagement metrics (likes, comments, shares, collections, and fan counts), video duration, and days since upload.

Uploader types were categorized into four groups: (1) Professional individuals (certified physicians); (2) Professional institutions (verified healthcare institutions, such as hospitals and Centers for Disease Control and Prevention); (3) Nonprofessional individuals (unverified individuals); (4) Nonprofessional institutions (unverified organizations).

In China, while prescription-based medical nutrition therapy is strictly regulated, general dietary advice can be disseminated by a range of sources, including certified physicians, registered dietitians, public dietitians, and even unverified social media creators. Reflecting this diverse landscape, professional background was further classified as cardiologists, gastroenterologists, other medical specialists (e.g., neurologists, traditional Chinese medicine physicians, nutritionists/dietitians, and emergency physicians), and self-media creators.

### 2.4 Content classification

Video content was categorized according to the framework of the Chinese Dietary Pagoda, which is a national guideline that organizes recommended food intake into hierarchical groups reflecting balanced nutrition (35). Specifically, five major food categories were defined: (1) Oil and salt: dietary fats and sodium intake, typically emphasized for restriction; (2) Dairy and soy products: milk, dairy derivatives, and plant-based proteins such as soy; (3) Animal-based foods: including meat, fish, eggs, and other protein sources; (4) Grains and tubers: staple foods such as rice, wheat, whole grains, and potatoes; (5) Fruits and vegetables: fresh produce rich in vitamins, fiber, and antioxidants. Each video was coded for the presence or absence of information related to these categories. Content comprehensiveness was evaluated as the number of these food groups covered within a single video.

### 2.5 Quality assessment

Video quality was evaluated using three validated instruments. The Global Quality Score (GQS) is a 5-point Likert scale assessing overall quality, flow, and usefulness of information (Supplementary Tables S1 and S2) (36). The modified DISCERN (mDISCERN) is a 5-item tool evaluating reliability, balance, and clarity of health information (Supplementary Tables S3 and S4) (37). The JAMA benchmark criteria (JAMA) is a 4-point scale assessing authorship, attribution, disclosure, and currency (Supplementary Tables S5 and S6) (38). Reviewers were trained prior to assessment to ensure consistency.

Two trained reviewers independently screened and evaluated all videos using a standardized coding manual. The protocol included predefined inclusion and exclusion criteria, definitions of content categories, and detailed instructions for applying the GQS, mDISCERN, and JAMA scoring tools. Prior to formal data extraction, both reviewers completed a pilot assessment to ensure consistency in interpretation. Any disagreements were resolved through consensus discussion. Furthermore, weighted Cohen's kappa showed substantial interrater agreement between the two reviewers for JAMA (*κ*w = 0.85), GQS (*κ*w = 0.82), and mDISCERN (*κ*w = 0.79).

### 2.6 Statistical analysis

Data were entered into a structured spreadsheet and checked for completeness and consistency. Each row represented one video, and each column represented one variable. Engagement metrics such as likes, comments, collections, shares, and fan counts were converted into numeric values. Video duration was converted into seconds, and time since upload was calculated in days. Dietary content categories were coded as binary variables, and content comprehensiveness was calculated as the number of dietary categories covered by each video, ranging from 1 to 5.

Continuous variables were summarized using medians and interquartile ranges due to non-normal distributions, while categorical variables were expressed as frequencies and percentages.

Radar charts were used to visually compare the distribution of dietary content categories across different uploader types and professional backgrounds. To evaluate differences in content comprehensiveness, the distribution of dietary categories across uploader types and professions was compared using Fisher's exact test.

The Kruskal–Wallis test was used to compare overall quality scores across uploader types and professional categories due to the ordinal nature of the outcomes and their non-normal distributions. Categories with sample sizes fewer than five were excluded from subgroup analyses to ensure statistical robustness and avoid unstable estimates. When significant differences were observed, *post hoc* pairwise comparisons were conducted using Dunn's test with Bonferroni correction. Violin plots were generated to visualize the distribution of quality scores across groups.

Spearman correlation analysis was used to examine associations between video quality scores and engagement metrics (fans, likes, comments, collections, shares), as well as correlations among GQS, mDISCERN, and JAMA scores to assess inter-tool consistency.

All analyses were performed using R software, with statistical significance defined as a two-sided *P* < 0.05.

## 3 Results

### 3.1 Video selection and basic characteristics

**Figure 1:**
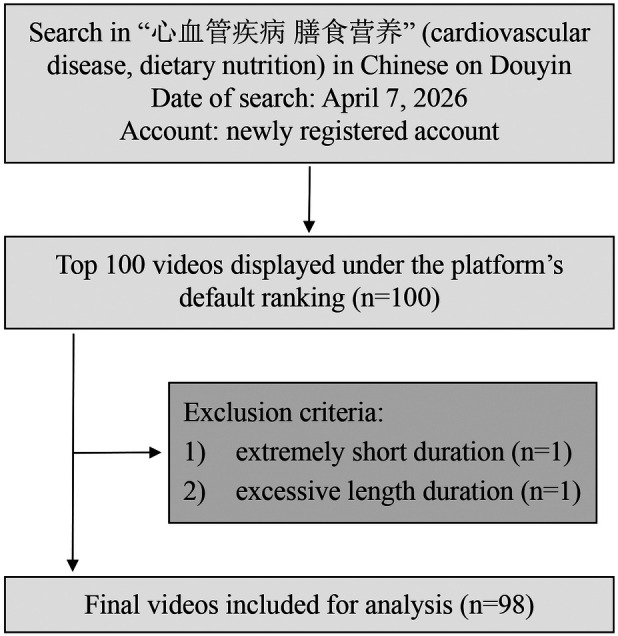
Flowchart of study selection and video screening process.

Among the first 100 retrieved videos, one video was excluded due to extremely short duration (12 s) and one due to excessive length (20 min). A total of 98 eligible videos were included in the final analysis (Figure 1). Among these, 59% were uploaded by professional individuals, followed by nonprofessional individuals (26%), professional institutions (11%), and nonprofessional institutions (4.1%).

Regarding professional background, cardiologists accounted for the largest proportion (32%), followed by self-media creators (30%), other specialists (29%), and gastroenterologists (10%).

The comprehensiveness of dietary content varied across videos. While 3 videos (3.1%) mentioned only one food group, the majority of videos covered multiple categories. Specifically, 22 videos (22%) mentioned two groups, 14 videos (14%) mentioned three groups, 32 videos (33%) mentioned four groups, and 27 videos (28%) covered all five food groups.

Engagement metrics demonstrated substantial variability, with median values of 11,000 for fans, 187 for likes, 8 for comments, 102 for collections, and 40 for shares. The median video duration was 55 s, and the median time since upload was 57 days (Table 1).

**Table 1:** Characteristics of dietary guidance for cardiovascular disease: related short videos.

Variable	*N* = 98
Uploaders	
Nonprofessional individuals	25 (26%)
Nonprofessional institutions	4 (4.1%)
Professional individuals	58 (59%)
Professional institutions	11 (11%)
Profession	
Cardiologists	31 (32%)
Gastroenterologists	10 (10%)
Other Specialists	28 (29%)
Self Media Creators	29 (30%)
Content Comprehensiveness	
1. food group	3 (3.1%)
2. food groups	22 (22%)
3. food groups	14 (14%)
4. food groups	32 (33%)
5. food groups	27 (28%)
Oil & Salt	46 (47%)
Dairy & Soy	59 (60%)
Animal Foods	66 (67%)
Fruits & Vegetables	96 (98%)
Grains & Tubers	85 (87%)
Fans	11,000 (5,587, 46,000)
Likes	187 (41, 697)
Comments	8 (3, 27)
Collections	102 (20, 441)
Shares	40 (10, 146)
Duration	55 (47, 71)
Days uploaded	57 (27, 104)

Data are presented as N (%) or median (Q1, Q3).

### 3.2 Content characteristics

Across all videos, the most frequently addressed category was fruits and vegetables (98%), followed by grains and tubers (87%) and animal-based foods (67%). In contrast, dairy and soy products (60%) and particularly oil and salt (47%) were less frequently discussed, indicating an imbalance in topic coverage (Table 1).

When mentioning fruits, vegetables, grains, and soy products, most videos advocated for increased consumption or appropriate intake. Conversely, oil and salt were generally identified as components to be restricted. Recommendations regarding animal-based foods were mixed: some videos suggested consuming fish, eggs, or lean meat, while others advised limiting fatty or processed meats.

**Figure 2:**
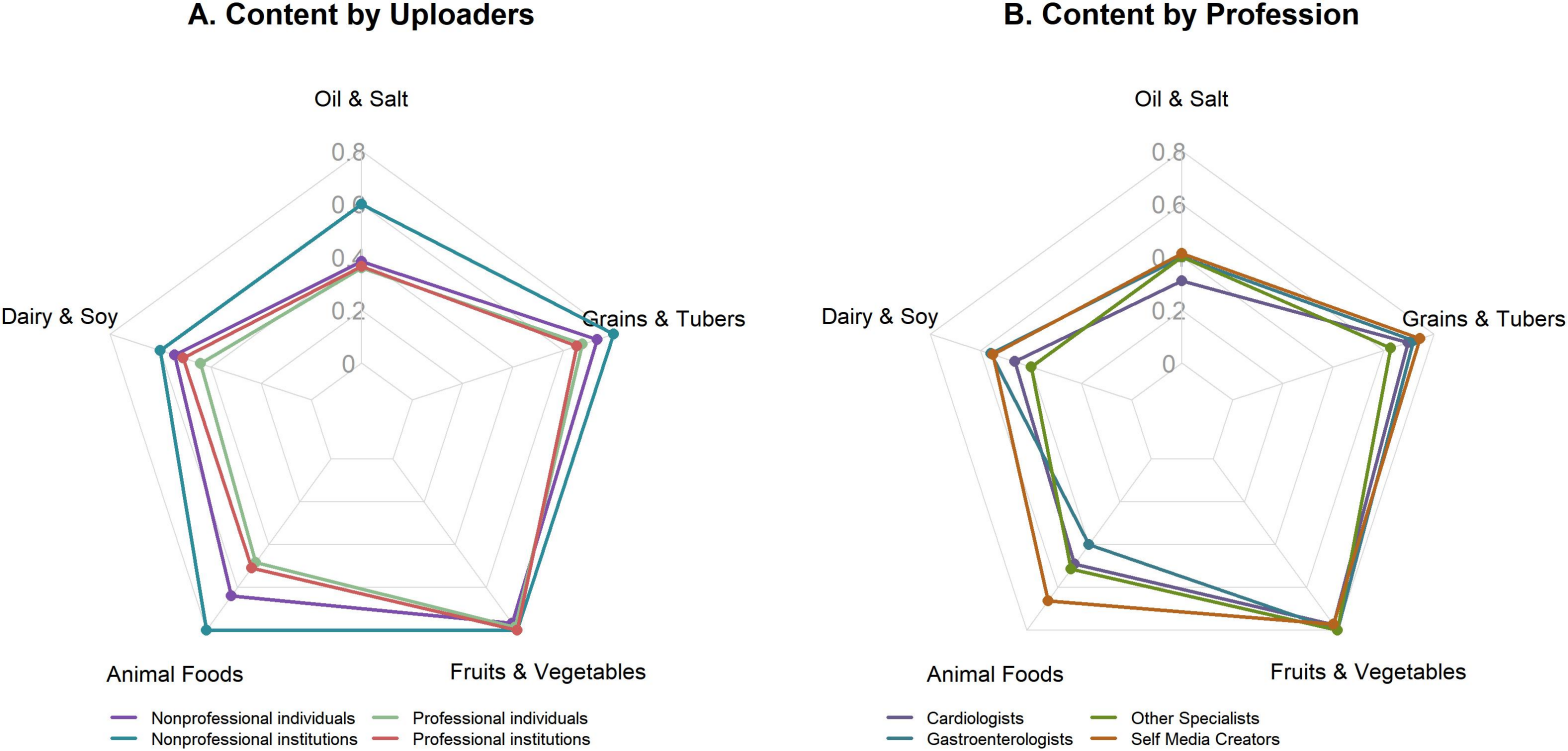
Comparison of content comprehensiveness stratified by uploaders and profession.

When stratified by uploader type, no statistically significant differences were observed in the distribution of dietary content categories (all *P* > 0.05; Supplementary Table S7). However, descriptive patterns suggested that professional and nonprofessional uploaders differed in emphasis. For example, nonprofessional institutions tended to cover animal-based foods more frequently, whereas professional individuals showed relatively lower coverage of oil and salt (Figure 2).

Similarly, no statistically significant differences were found across professional backgrounds (all *P* > 0.05; Supplementary Table S8). Nevertheless, observable trends indicated that self-media creators more frequently focused on animal-based foods, while medically trained professionals tended to present a more balanced distribution across food categories (Figure 2).

### 3.3 Distribution of video quality scores

**Figure 3:**
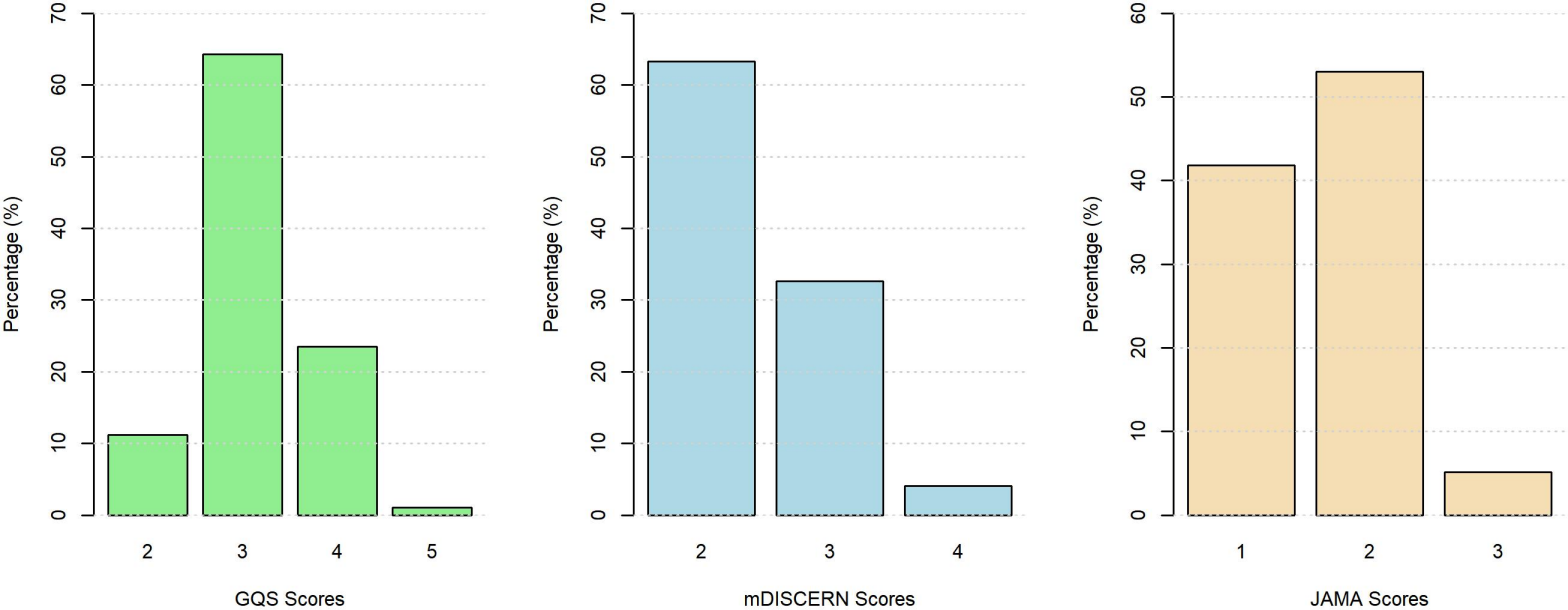
Distribution of three evaluation tools.

Overall, video quality was moderate. The majority of videos were rated as GQS score 3 (64%), followed by score 4 (23%). For mDISCERN, most videos scored 2 (63%) or 3 (33%), indicating limited reliability. JAMA scores were generally low, with 53% scoring 2 and only a small proportion achieving higher credibility levels (Figure 3 and Supplementary Table S9).

### 3.4 Comparison of video quality

**Figure 4:**
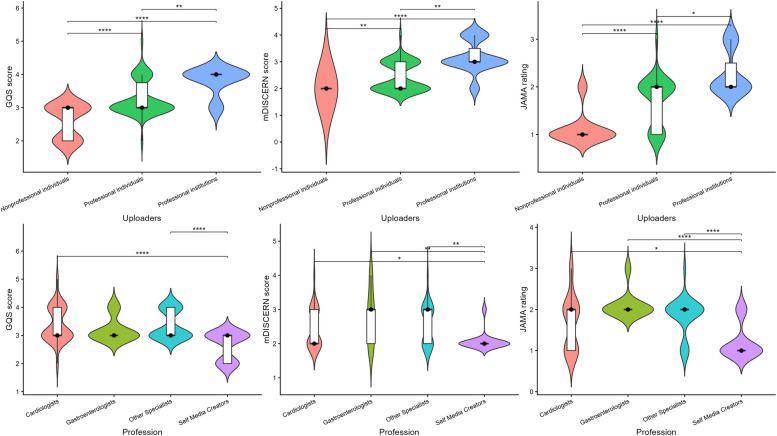
Comparison of video quality scores stratified by uploaders and profession. Box plots illustrate the score distribution for each subgroup. For categories containing 5 or more observations, the Kruskal–Wallis test was applied to test the differences in overall quality scores. When significant differences were observed, *post hoc* pairwise comparisons were conducted using Dunn's test with Bonferroni correction. The central horizontal line denotes the median, and the whiskers extend to the interquartile range (IQR). * *P* < 0.05, ** *P* < 0.01, *** *P* < 0.005, **** *P* < 0.001.

Significant differences in video quality were observed across uploader categories (Figure 4). Videos produced by professional institutions achieved the highest scores across all three instruments, followed by professional individuals, while nonprofessional individuals consistently had the lowest scores (all *P* < 0.001; Supplementary Table S10).

Similarly, quality varied by professional background. Videos created by cardiologists, gastroenterologists, and other medical specialists demonstrated higher reliability and overall quality compared with those produced by self-media creators, which showed consistently lower scores across GQS, mDISCERN, and JAMA (all *P* ≤ 0.001; Supplementary Table S1^1^).

### 3.5 Correlation between video quality and engagement metrics

**Figure 5:**
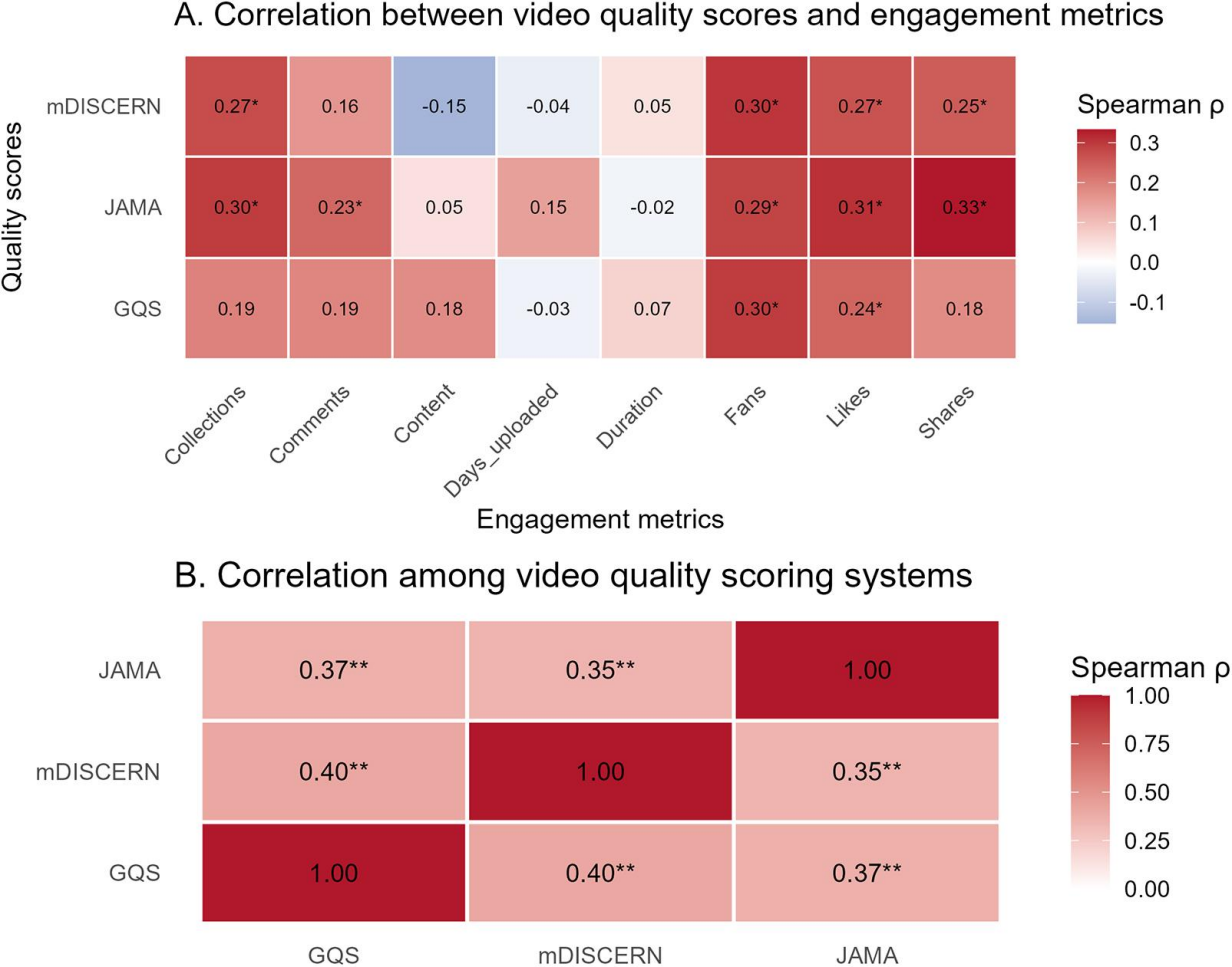
Heatmaps of correlations between video quality scores and engagement metrics and among the video quality scores. * *P* < 0.05, ** *P* < 0.01.

Correlation analysis revealed weak but statistically significant positive associations between quality scores and certain engagement indicators, particularly fan counts and likes (Figure 5A and Supplementary Table S12). However, correlations with video duration and days since upload were not significant. In contrast, moderate positive correlations were observed among the three quality assessment tools themselves (Figure 5B and Supplementary Table S13), supporting internal consistency across evaluation instruments.

## 4 Discussion

### 4.1 Principal findings

In this comprehensive cross-sectional study, we evaluated the quality and reliability of Douyin videos related to dietary guidance for CVD using the modified DISCERN instrument (mDISCERN), Global Quality Score (GQS), and JAMA benchmark criteria. Overall, these short-form videos were brief in duration and demonstrated an imbalanced thematic coverage. Most content focused on fruits, vegetables, and grains, whereas key dietary components such as salt and oil restriction were markedly underrepresented. The overall quality and reliability of the videos were moderate to low. Stratified analyses further revealed significant heterogeneity across uploader types, with videos produced by cardiologists and other healthcare professionals achieving higher quality scores. Additionally, weak positive correlations were observed between video quality and certain engagement metrics, whereas no significant associations were found with video duration or time since upload. These findings underscore the need to enhance both the professionalism and comprehensiveness of CVD-related dietary information disseminated via short-video platforms and provide important implications for platform governance and evidence-based content production strategies.

### 4.2 Video content, quality, and reliability

Our findings indicate that although 61% of videos covered four or five food groups, there was a substantial imbalance in the thematic distribution of dietary content in CVD-related videos. While fruits, vegetables, grains, and animal-based foods were frequently mentioned, critical recommendations regarding sodium and fat restriction were notably scarce. This pattern suggests that current Douyin content tends to prioritize visually appealing and cognitively intuitive food categories, while neglecting less salient but clinically crucial dietary restrictions. However, excessive sodium and fat intake are well-established modifiable risk factors for hypertension and coronary heart disease (39, 40). Prior evidence has demonstrated a strong positive association between high sodium intake and cardiovascular events (41), with the Global Burden of Disease (GBD) 2019 study attributing approximately 1.715 million CVD-related deaths to high sodium diets (42). Similar trends have been reported on other social media platforms. For instance, Sadip et al. found that YouTube videos on acute myocardial infarction tended to emphasize general, easily disseminated health advice while rarely addressing high-risk behavioral interventions (43).

In our subgroup analyses, non-professional creators were more likely to emphasize animal-based foods, whereas even professional institutions demonstrated insufficient coverage of sodium and fat restriction. This phenomenon may be attributable to the underlying content amplification mechanisms of social media platforms, where algorithms preferentially promote emotionally engaging and entertainment-oriented content (44–46). From a health communication perspective, prolonged exposure to content emphasizing “beneficial” foods while downplaying restrictive dietary components may lead to distorted perceptions of optimal dietary patterns among patients. Therefore, both platforms and content creators should strive for a balanced presentation of dietary recommendations, incorporating evidence-based guidance—such as salt reduction, fat control, and balanced nutrient intake—into video narratives or thematic tags to promote comprehensive understanding.

Using mDISCERN, GQS, and JAMA criteria, we found that only 24.0% of videos achieved a GQS ≥4, while 63.0% scored 2 on the mDISCERN scale, indicating generally suboptimal quality. These findings are consistent with previous studies assessing online video content related to weight management and inflammatory bowel disease dietary guidance (47, 48). As a “fragmented information medium” (49, 50), short videos are inherently constrained by limited duration (median: 55 s in this study), which hinders the effective communication of complex medical information. Moreover, Douyin's algorithmic preference for engaging content may incentivize creators to prioritize entertainment over educational value, potentially compromising informational accuracy and depth.

### 4.3 Differences in video quality across uploader types

Significant differences in video quality were observed across uploader categories. Content produced by healthcare professionals and medical institutions consistently achieved higher quality scores compared to that generated by non-professional users. This trend aligns with findings from prior studies in other health domains (51–53), reinforcing the importance of professional expertise in ensuring content accuracy and reliability. Healthcare professionals, particularly cardiologists, are more likely to provide structured, evidence-based information grounded in clinical guidelines and consensus statements. However, it is noteworthy that even videos produced by physicians did not consistently meet high-quality standards. This suggests that medical expertise alone does not guarantee effective health communication in digital environments. Several factors may explain this discrepancy, including platform-specific dissemination logic, algorithmic preferences, and format constraints such as brevity and visual engagement requirements (54–56). Physicians may face challenges in balancing accuracy with conciseness, as simplifying complex medical information to fit short-video formats risks omitting critical details. These findings highlight the need for targeted training programs that equip healthcare professionals with skills in digital health communication, including concise messaging, audience engagement strategies, and visual storytelling techniques.

### 4.4 Association between engagement metrics and video quality

Previous studies have identified positive associations between engagement metrics and video quality (32, 57–60). In our analysis, although likes and follower counts were positively correlated with quality scores, these relationships were weak. This suggests that while higher-quality videos may be more likely to gain user recognition, their dissemination is also heavily influenced by platform algorithms and the immediate appeal of the content. Importantly, high popularity does not necessarily equate to high informational accuracy or reliability. Therefore, relying solely on engagement metrics to evaluate content quality is insufficient. Platforms and content creators should collaborate to enhance users' ability to critically appraise online health information, establish standardized content guidelines, and implement professional oversight mechanisms. Interestingly, no significant associations were found between video quality and either video duration or time since upload, indicating that informational value, rather than temporal or structural characteristics, is the primary determinant of quality. Additionally, moderate positive correlations were observed among the three evaluation tools, consistent with previous findings (61), supporting their validity and methodological robustness in assessing short-form health video content.

### 4.5 Strengths, limitations, and future directions

This study is the first to employ multiple complementary evaluation instruments—Global Quality Scale (GQS), modified DISCERN (mDISCERN), and Journal of the American Medical Association (JAMA) benchmarks—to assess the quality of diet-related cardiovascular disease (CVD) content on Douyin. The combined use of these tools enables a multidimensional appraisal of video quality, encompassing information coverage, credibility of treatment options, reliability of content, and overall quality. Furthermore, this study systematically explored the associations between video engagement metrics (including likes, comments, favorites, and shares) and content quality, as well as the interrelationships among GQS, mDISCERN, and JAMA scores. These correlation analyses enhance the robustness and practical relevance of our findings. Our results suggest that platforms such as Douyin could benefit from collaboration with professional healthcare organizations, and that increasing the involvement of qualified healthcare professionals in content creation may substantially improve the quality of diet-related CVD information. Nevertheless, several limitations should be acknowledged. First, only Chinese-language videos from the Douyin platform were included, which may limit the generalizability of our findings to other linguistic contexts (e.g., English) and social media platforms (e.g., YouTube, Bilibili, and Xiaohongshu). Second, the relatively small sample size (*n* = 98) and cross-sectional design capture only a snapshot in time, precluding assessment of temporal trends in content quality and potentially limiting the timeliness and external validity of the findings. Third, the evaluation of quality and reliability relied on GQS, mDISCERN, and JAMA scoring systems; although widely used, these instruments inherently involve a degree of subjectivity. Future research should expand sample sizes, include multiple short-video platforms across diverse linguistic and cultural settings, and adopt longitudinal designs to track changes in content quality over time. Additionally, the integration of more objective, automated assessment tools may further strengthen the validity and reproducibility of findings. Fourth, the analysis focused primarily on dietary components. Future research incorporating comprehensive thematic analysis would provide a more holistic understanding of the health narratives presented in short-form videos.

With the rapid growth of internet-based health promotion, our findings carry important implications for short-video dissemination and public health education. There is an urgent need to establish standardized criteria for health-related content published on social media platforms and to improve public awareness of information quality. Regulatory authorities and platform operators should strengthen oversight of health-related content; for example, policies in China already require health content creators to verify their professional credentials (62). Similar regulatory efforts and platform governance strategies are also emerging globally across major short-video platforms, including TikTok, Instagram Reels, and YouTube Shorts, which is consistent with findings from recent international studies on short-video-based health communication (17, 49). However, the level of implementation and enforcement varies considerably across countries and regulatory environments. This highlights a shared global challenge in balancing rapid information dissemination with the need to ensure the accuracy and reliability of online health content. Healthcare professionals should actively leverage these platforms to design, produce, and disseminate high-quality, evidence-based content that is both scientifically rigorous and accessible to the general public. Collaborative initiatives between professional institutions and platforms could facilitate the development of authoritative educational series, accompanied by clear in-app labeling to help users identify credible sources. Importantly, while these recommendations are grounded in the Chinese digital health communication context, they have broader implications for global public health practice. As short-video platforms increasingly function as primary channels for health information worldwide, strengthening content governance, professional engagement, and public digital health literacy is essential across diverse sociocultural and regulatory settings to mitigate misinformation risks and improve population health outcomes.

## 5 Conclusion

This study evaluated the informational quality of 98 short videos related to CVD dietary guidance on the Douyin platform. The findings indicate that the overall quality of CVD-related dietary content remains suboptimal, with notable imbalances in thematic coverage. Furthermore, video quality was significantly influenced by the type of content uploader. Videos produced by professional organizations demonstrated superior comprehensiveness, quality, and reliability compared to those created by non-professional individuals. In addition, dietary recommendations delivered by cardiologists and other medical experts were more informative and evidence-based than those provided by independent content creators.

Overall, given the rapid proliferation of video-sharing platforms, there is an urgent need to implement professional content regulation and targeted educational interventions, as well as to establish standardized criteria for evaluating the quality of health-related information.

## Data Availability

The raw data supporting the conclusions of this article will be made available by the authors, without undue reservation.
