# Supplementary material for: The reliability and quality of short videos as a source of dietary guidance for cardiovascular disease: a cross-sectional study
Source: Front Cardiovasc Med. 2026 Jul 29;13:1878079. doi: 10.3389/fcvm.2026.1878079 (PMC13461494; doi:10.3389/fcvm.2026.1878079)
Supplement: Supplementary file 1 [file Datasheet1.docx]

**Supplementary table S1. Global Quality Scale (GQS)-Original English Version.**

| **GQS Definition** | **Score** |
| --- | --- |
| Poor quality: Specifically, the content is illogical, the mobility is poor, most of the information is missing, and it is useless for patients. | 1 |
| Generally poor quality: the content logic is poor, although some information is listed, more important information is still missing, and the use of patients is very limited. | 2 |
| Moderate quality: some important information is adequately discussed. | 3 |
| Good quality and flow: Specifically, the video logic is clear and smooth, covering most of the relevant information, which is useful for patients. | 4 |
| Excellent quality and flow: Specifically, the video logic is clear, and the content is very smooth, which is very useful for patients. | 5 |

**Supplementary table S2. Global Quality Scale (GQS)-Chinese Translated Version.**

| **定义** | **得分** |
| --- | --- |
| **质量较差**：内容缺乏逻辑性，连贯性差，缺失大部分关键信息，对患者无参考价值。 | 1 |
| 质量一般：内容逻辑欠佳，虽列举了部分信息，但仍缺失较多重要内容，对患者的参考价值十分有限。 | 2 |
| 质量中等：涉及了部分重要信息，并进行了适当阐述。 | 3 |
| 质量良好，逻辑通畅：视频逻辑清晰流畅，涵盖了大部分相关信息，对患者具有较高的实用价值。 | 4 |
| 质量优秀，逻辑极佳：视频逻辑缜密，内容呈现行云流水，对患者极具参考价值。 | 5 |

**Supplementary table S3. Modified DISCERN (mDISCERN)-Original English Version.**

| **Reliability Score** | **Score** |
| --- | --- |
| 1. Is the video clear, concise, and understandable? | 1 point for answer ‘yes’,  0 point for answer ‘no’ |
| 2. Are valid sources cited? |  |
| 3. Is the content presented balanced and unbiased? |  |
| 4. Are additional sources of content listed for patient reference? |  |
| 5. Are areas of uncertainty mentioned? |  |

**Supplementary table S4. Modified DISCERN (mDISCERN)-Chinese Translated Version.**

| **Reliability Score** | **Score** |
| --- | --- |
| 1. 视频内容是否清晰、简明且易于理解？ | 回答“是”得 1分；  回答“否”得 0分。 |
| 2. 是否引用了有效的信息来源？ |  |
| 3. 内容呈现是否客观、公正且无偏倚？ |  |
| 4. 是否为患者列出了可供参考的其他信息来源？ |  |
| 5. 是否提及了存在不确定性的领域？ |  |

**Supplementary table S5. The Journal of the American Medical Association (JAMA) Benchmarks – Original English Version.**

| Score* | Score component | |
| --- | --- | --- |
| 1 score | Authorship | Author and contributor credentials and their affiliations should be provided. |
| 1 score | Attribution | Clearly lists all copyright information and states references and sources for content. |
| 1 score | Currency | Initial date of posted content and subsequent updates to content should be provided. |
| 1 score | Disclosure | Conflicts of interest, funding, sponsorship, advertising, support, and video ownership  should be fully disclosed. |

*The criteria of each aspect were scored separately, and 1 point for each criterion with a total score of 4 points.

**Supplementary table S6. The Journal of the American Medical Association (JAMA) Benchmarks – Chinese Translated Version.**

| 得分* | 评分细则 | |
| --- | --- | --- |
| 1 分 | 作者署名 | 应提供作者及贡献者的资质证明及其所属机构信息。 |
| 1 score | 归属来源 | 应清晰列出所有版权信息，并注明内容的参考文献与资料来源。 |
| 1 score | 时效性 | 应提供内容的发布日期及后续的更新日期。 |
| 1 score | 利益披露 | 应充分披露利益冲突、资助方、赞助商、广告支持、资金援助及视频所有权归属。 |

*各项指标分别评分，每项符合得 1 分，总分 4 分。

**Supplementary table S7. Content distribution by Uploaders.**

| **Variable** | Nonprofessional individuals (n=25) | Nonprofessional institution (n=4) | Professional individuals (n=58) | Professional institution (n=11) | P for Fisher’s exact test |
| --- | --- | --- | --- | --- | --- |
| **Oil & Salt** | 12 (48.0%) | 3 (75.0%) | 26 (44.8%) | 5 (45.5%) | 0.763 |
| **Dairy & Soy** | 17 (68.0%) | 3 (75.0%) | 32 (55.2%) | 7 (63.6%) | 0.667 |
| **Animal Foods** | 20 (80.0%) | 4 (100.0%) | 35 (60.3%) | 7 (63.6%) | 0.185 |
| **Fruits & Vegetables** | 24 (96.0%) | 4 (100.0%) | 57 (98.3%) | 11 (100.0%) | 0.652 |
| **Grains & Tubers** | 23 (92.0%) | 4 (100.0%) | 49 (84.5%) | 9 (81.8%) | 0.747 |

**Supplementary table S8. Content distribution by Profession.**

| **Variable** | Cardiologists (n=31) | Gastroenterologists (n=10) | Other Specialists (n=28) | Self-Media Creators (n=29) | P for Fisher’s exact test |
| --- | --- | --- | --- | --- | --- |
| **Oil & Salt** | 12 (38.7%) | 5 (50.0%) | 14 (50.0%) | 15 (51.7%) | 0.754 |
| **Dairy & Soy** | 18 (58.1%) | 7 (70.0%) | 14 (50.0%) | 20 (69.0%) | 0.470 |
| **Animal Foods** | 19 (61.3%) | 5 (50.0%) | 18 (64.3%) | 24 (82.8%) | 0.143 |
| **Fruits & Vegetables** | 30 (96.8%) | 10 (100.0%) | 28 (100.0%) | 28 (96.6%) | 1.000 |
| **Grains & Tubers** | 27 (87.1%) | 9 (90.0%) | 22 (78.6%) | 27 (93.1%) | 0.452 |

**Supplementary table S9. Distribution of three evaluation tools.**

| **Evaluation tools** | Score | n (%) |
| --- | --- | --- |
| **GQS** | 2 | 11 (11%) |
|  | 3 | 63 (64%) |
|  | 4 | 23 (23%) |
|  | 5 | 1 (1.0%) |
| **mDISCERN** | 2 | 62 (63%) |
|  | 3 | 32 (33%) |
|  | 4 | 4 (4.1%) |
| **JAMA** | 1 | 41 (42%) |
|  | 2 | 52 (53%) |
|  | 3 | 5 (5.1%) |

**Supplementary table S10. Comparison of video quality scores stratified by uploader category with n≥5.**

| **Scores** | Nonprofessional individuals (n=25) | Professional individuals (n=58) | Professional institution (n=11) | Kruskal-Wallis H | P |
| --- | --- | --- | --- | --- | --- |
| GQS, Median (Q1, Q3) | 3 (2, 3) | 3 (3, 4) | 4 (4, 4) | 35.3 | <0.001 |
| mDISCERN, Median (Q1, Q3) | 2 (2, 2) | 2 (2, 3) | 3 (3, 4) | 31.1 | <0.001 |
| JAMA, Median (Q1, Q3) | 1 (1, 1) | 2 (1, 2) | 2 (2, 3) | 34.0 | <0.001 |

**Supplementary table S11. Comparison of video quality scores stratified by profession with n≥5.**

| **Scores** | Cardiologists (n=31) | Gastroenterologist (n=10) | Other Specialists (n=28) | Self-Media Creators (n=29) | Kruskal–Wallis H | P |
| --- | --- | --- | --- | --- | --- | --- |
| GQS, Median (Q1, Q3) | 3 (3, 4) | 3 (3, 3) | 3 (3, 4) | 3 (2, 3) | 27.0 | <0.001 |
| mDISCERN, Median (Q1, Q3) | 2 (2, 3) | 3 (2, 3) | 3 (2, 3) | 2 (2, 2) | 17.5 | 0.001 |
| JAMA, Median (Q1, Q3) | 2 (1, 2) | 2 (2, 2) | 2 (2, 2) | 1 (1, 1) | 28.5 | <0.001 |

**Supplementary table S12.** **Spearman correlation between video quality scores and engagement metrics.**

| video quality scores | engagement metrics | Spearman's ρ | *P* | *P*_adj_ |
| --- | --- | --- | --- | --- |
| GQS | Content | 0.179 | 0.077 | 0.124 |
|  | Fans | 0.296 | 0.003 | **0.015** |
|  | Likes | 0.238 | 0.018 | **0.043** |
|  | Comments | 0.188 | 0.064 | 0.117 |
|  | Collections | 0.195 | 0.054 | 0.109 |
|  | Shares | 0.18 | 0.076 | 0.124 |
|  | Duration | 0.068 | 0.506 | 0.639 |
|  | Days_uploaded | -0.027 | 0.794 | 0.822 |
| mDISCERN | Content | -0.153 | 0.133 | 0.187 |
|  | Fans | 0.304 | 0.002 | **0.015** |
|  | Likes | 0.265 | 0.008 | **0.025** |
|  | Comments | 0.163 | 0.109 | 0.163 |
|  | Collections | 0.274 | 0.006 | **0.022** |
|  | Shares | 0.252 | 0.012 | **0.033** |
|  | Duration | 0.045 | 0.658 | 0.752 |
|  | Days_uploaded | -0.035 | 0.732 | 0.798 |
| JAMA | Content | 0.047 | 0.644 | 0.752 |
|  | Fans | 0.285 | 0.004 | **0.018** |
| video quality scores | engagement metrics | Spearman's ρ | P | **P**adj |
|  | Likes | 0.31 | 0.002 | **0.015** |
| JAMA | Comments | 0.235 | 0.020 | **0.043** |
|  | Collections | 0.297 | 0.003 | **0.015** |
|  | Shares | 0.333 | 0.001 | **0.015** |
|  | Duration | -0.023 | 0.822 | 0.822 |
|  | Days uploaded | 0.147 | 0.147 | 0.197 |

*P*_adj_ were adjusted for multiple testing using the false discovery rate (FDR) method. Statistical significance is defined as *P*_adj_ < 0.05.

**Supplementary table S13.** **Spearman correlation between different video quality scores.**

| Scores 1 | Score 2 | Spearman's ρ | *P* | *P*_adj_ |
| --- | --- | --- | --- | --- |
| GQS | mDISCERN | 0.405 | <0.001 | <0.001 |
| GQS | JAMA | 0.369 | <0.001 | 0.001 |
| mDISCERN | JAMA | 0.348 | <0.001 | 0.001 |

*P*_adj_ were adjusted for multiple testing using the false discovery rate (FDR) method. Statistical significance is defined as *P*_adj_ < 0.05.
